# Evaluation of a performance enhancing supplement in American Foxhounds during eventing*

**DOI:** 10.1017/jns.2014.38

**Published:** 2014-09-25

**Authors:** Janice L. Huntingford, Brent N. Kirn, Kerry Cramer, Sabine Mann, Joseph J. Wakshlag

**Affiliations:** 1 Essex Animal Hospital, Essex, Ontario, Canada; 2 Trouw Nutrition USA, 115 Executive Dr Highland, IL, USA; 3 Department of Population Medicine, Cornell University, College of Veterinary Medicine, Ithaca, NY, USA; 4 Department of Clinical Sciences, Cornell University, College of Veterinary Medicine, Ithaca, NY, USA

**Keywords:** Performance, American Foxhounds, Creatine kinase, Exercise, Endurance, AST, aspartate aminotransferase, CBC, complete blood count, CK, creatine kinase

## Abstract

Enhancing performance through dietary measures is constantly sought as some supplements have shown modest performance enhancement in rodents and human subjects. To evaluate a proprietary dietary supplement, a study was undertaken to assess the effect of diet and exercise on blood physiological parameters during a tracking American Fox Hound field championship. Ten dogs were assigned to two different groups. Group A received a commercial kibble and Group B received the same diet with the addition of a supplement added to the dietary premix for 4 weeks before the field event. Blood was collected at rest, immediately following days 1 and 2 of the event and 48 h after day 2. Blood chemistry, complete blood cell counts and cortisol concentrations were analysed. Competition performance was also documented for all dogs using a points system for tracking events. Many chemistry parameters and blood cell counts changed significantly due to exercise. Differences between the dietary groups showed that Group B had significantly lower aspartate aminotransferase on days 1 and 2 of exercise and lower creatine kinase on day 2. Based on tracking scores, dogs in Group B out-performed dogs in Group A. This study suggests that endurance hunting dogs develop changes in serum markers of musculoskeletal integrity that might be mitigated by the addition of the supplement, resulting in better performance. Although intriguing, follow-up controlled studies are needed to ensure that the enhanced performance was not biased due to lack of randomisation.

According to the American Kennel Club, organised events for dogs and their handlers have grown significantly. Performance tests such as field trials, hunt tests, lure coursing and coonhound events attracted 240 000 entries in 2011 making hunting performance dogs a significant group of canine athletes (http://www.akc.org/about/annual_report.cfm). Hunting dogs are required to perform low-intensity tracking activity over several hours, interspersed with short bursts of sprinting. The intensity of the work done by the dog is highly variable throughout the hunt^(^^1^^)^. Although there have been a number of papers published on racing Greyhounds and sled dogs, the current literature contains little information on the performance hunting dog as an athlete^(^^1^^–^^3^^)^, with no information on the physiological and biochemical changes that occur due to the stress of the event.

Enhancing performance in the canine athlete through dietary measures has been constantly sought as some supplements have shown modest performance enhancement in rodents and human subjects^(^^4^^–^^7^^)^. A number of studies involving sled dogs and racing Greyhounds have been published showing that dietary supplements enriched with antioxidants such as vitamins E, C and β-carotene fail to ameliorate rises in creatine kinase (CK) as a marker of muscle permeability changes^(^^8^^–^^10^^)^. Conversely, sled dogs that had high-serum vitamin E concentrations prerace had a higher chance of finishing an endurance race than those with low-serum vitamin E concentrations; however, this may be a reflection of overall diet rather than vitamin E specifically^(^^11^^)^.

Antioxidants, such as vitamins E and C may, in fact, have detrimental effects on performance in racing Greyhounds^(^^12^^,^^13^^)^. Although these vitamins and antioxidants have not proven particularly useful, the effects of compounds such as betaine during physical heat stress and resveratrol for improved mitochondrial function in rodents and human subjects hold promise^(^^4^^–^^7^^)^.

It has been established that athletic dogs undergo oxidative and thermal stress during exercise^(^^2^^,^^3^^,^^9^^,^^10^^,^^14^^)^. Betaine supplementation in human subjects has been hypothesised to enhance sports performance through maintenance of thermal homoeostasis and improved cellular hydration during exercise in hot environments^(^^15^^)^. l-Carnitine supplementation has been shown to have a protective effect against exercise-induced oxidative stress in rats^(^^16^^)^. The aim of our study was to evaluate the effect of a dietary proprietary supplement containing betaine, l-carnitine, dietary buffers, B-vitamins and yeast extract antioxidants on coyote tracking American Foxhounds during an event through evaluation of blood chemistry, complete blood cell counts (CBC) and cortisol concentrations.

## Experimental methods

All procedures were approved by the Cornell University Institutional Animal Care and Use Committee. Ten healthy American Foxhounds between the ages of 2 and 6 years old (four females and six males) from two kennels were used in the present study. All dogs in the study were fed between 220 and 300 g of a commercial kibble containing 22 % fat and 31 % crude protein, 3 % crude fibre and 8 % moisture (see Supplementary Table 1 for complete nutrient profile). Five dogs were provided a diet that had proprietary blend of yeast, betaine, magnesium, l-carnitine, sodium bicarbonate, biotin, niacin, pantothenic acid, pyridoxine and cyanocobalamin in descending order which was incorporated into the kibble pre-extrusion. The other five dogs were fed the base food without the additional ingredients as controls. Dogs were from two kennels (four dogs from one kennel and six dogs from another) and were randomly fed based on the group housing with a treatment diet and control diet group in each kennel (two dogs in each of two pens at kennel 1 and three dogs in each of two pens in kennel 2). All dogs were fed the diets for 4 weeks before testing. All ten dogs competed in the 3-d South Carolina Field Championship in Manning, South Carolina in a 2000-acre area. The competition was approximately 5 h daily in an average high ambient temperature of 19·6°C (range 13·9–26·7°C). Based on GPS collar readings on one competitive dog (in the dietary supplement group), the average speed was 8·2 km/h for the duration of the trial.

**Table 1.** Median and ranges of complete blood count results expressed at rest, after days 1 and 2 of competition, and 48 h after competition in the South Carolina Field Championship in control and supplemented dogs (*n* 5 in each group). Reference ranges are displayed in the left-hand column in parentheses

CBC (ref. range)	Group	Resting	Day 1	Day 2	48 h post comp
RBC (4·8–9·3 mill/ml)	Control	5·6 (5·2–6·2)	5·8 (5·2–6·0)	6·2 (5·4–6·6)	5·6 (5·3–6·0)
	Test	5·9 (3·6–6·9)	5·6 (3·4–6·8)	5·6 (3·7–6·9)	5·5 (3·5–6·5)
Haemoglobin (12·1–20·3 g/dl)	Control	12·3 (11·9–12·6)	12·5 (11·8–13·3)	13·2 (12·1–14·0)^a^	12·1 (11·2–12·7)
	Test	11·3 (8·1–12·5)	11·3 (7·8–13·2)	11·2 (8·5–13·1)	11·3 (8·0–12·9)
Haematocrit (36–60 %)	Control	41 (41–42)	42 (39–43)	42 (38–45)	40 (38–42)
	Test	38 (27–42)	38 (26–42)	37 (28–43)	37 (25–42)
MCV (58–79 fl)	Control	74 (69–79)	73 (71–76)	69 (64–72)^a^	71 (68–74)
	Test	66 (56–75)	69 (63–79)	68 (62–78)	68 (60–75)
MCH (19–28 pg)	Control	22 (20–23)	22 (20–23)	22 (21–23)	22 (20–23)
	Test	19 (17–22)	20·36 (17–23)	20 (18–23)	21 (18–23)
MCHC (30–38 g/dl)	Control	29 (29–30)	30 (28–31)	31 (32–32)	30 (29–31)
	Test	30 (29–31)	30 (28–32)	30 (28–32)	30 (30–32)
Platelets (170–400 thou/ml)	Control	292 (206–341)	296 (209–444)	321 (231–387)	299 (262–372)
	Test	315 (235–360)	265 (176–326)	322 (217–371)	383 (258–456)
Erythrocytes count (thou/ml) (4·0–14·5)	Control	12·9 (9·3–16·5)	25·7 (17·6–36·8)^a^	16·8 (14·2–22·6)	14·3 (10·3–21·1)
	Test	12·5 (7·1–17·3)	22·0 (12·8–29·5)^a^	13·8 (10·4–18·2)	12·3 (8·7–16·0)
Neutrophils (2060–10 600/µl)	Control	8234 (4005–12 375)	22 318 (14 960–32 384)^a^	13 571 (11 644–18 080)^a^	9097 (6099–15 192)
	Test	8276 (4047–12 696)	21 096 (10 496–36 550)^a^	11 320 (8840–14 742)	8408 (5916–11 360)
Lymphocytes (690–4500/µl)	Control	2760 (2475–3160)	2145 (1528–3312)	2167 (1704–3164)	2912 (2247–4350)
	Test	2533 (1846–3531)	1767 (1288–2259)	1529 (1040–2366)^a^	2116 (1479–2730)
Monocytes (0–840/µl)	Control	672 (534–948)	295 (176–382)^a^	581 (426–904)	995 (515–1688)
	Test	771 (355–1104)	420 (184–753)^a^	564 (294–768)	1189 (460–2720)
Eosinophils (0–1200/µl)	Control	1274 (465–2370)	923 (538–1680)	432 (284–652)^a^	1336 (584–2400)^a,b^
	Test	974 (321–1242)	697 (384–1288)	296 (0–735)^a^	567 (0–1170)^a,b^

^a^Indicates a significant difference from resting within group (*P* < 0·05).

^b^Significant difference between treatment groups (*P* < 0·05).

Ten millilitres of blood was collected from the jugular vein of each dog with a 21-gauge needle and transferred to a 3-ml tube containing EDTA and a 7-ml tube red top tube. Blood was collected at four different time points: a resting sample 24 h before the competition, a post-race sample on day 1 within 30 min of completion of exercise, a post-race sample on day 2 within 30 min of completion of exercise and a sample 48 h after the end of the competition. Serum was separated within 1 h of collection by centrifugation at 4000 ***g*** for 6 min and was transferred to a separate tube. Both EDTA and serum samples were stored at 4°C during overnight transport to the laboratory (Antech Diagnostics Inc).

CBC results were obtained using Bayer ADVIA 120 analyzer (Siemens Corp). Serum biochemical analytes (sodium, chloride, potassium, glucose, total protein, globulin, creatinine, urea, cholesterol, total bilirubin, alanine aminotransferase, aspartate aminotransferase (AST), amylase, lipase and CK) were analysed using a Olympus AU5400 automated analyser (Olympus America).

### Hunt performance

A total of 426 dogs were entered in the event. All dogs in the trial were rated by judges in the field for four categories, including endurance, speed, tracking and overall performance; dogs could place within the top ten in any one of these categories. If placing in the top ten in any of these categories a dog was awarded one point, with the potential for a total of four points. Therefore each group could accumulate a total of twenty points.

### Statistical analysis

All CBC and chemistry values were statistically assessed using repeated measures analysis using PROC MIXED in SAS (v. 9.3, SAS Institute Inc) with the treatment group and time as fixed effects as well as their interaction. Since measurements were unequally spaced in time, covariance structures tested included spatial (power law, Gaussian and spherical) as well as unstructured and compound symmetry. Inference on the parameter in question was made only when the assumption of normality of residuals was met and the model with the lowest Aikaike Information Criteria was chosen if several models could be fitted. Differences from baseline values were assessed using *post hoc* Tukey's HSD accounting for multiple comparisons Statistical significance was defined as *P* < 0·05. To assess performance in the field a Fisher's exact test was used to examine point differences between the control and test diet groupings.

Contingency tables set up with a total of twenty possible points for each group.

## Results

### Complete blood count

CBC results showed that MCV and haemoglobin were significantly different (MCV – lower and haemoglobin – higher) than resting on day 2 of exercise in the control group (Table 1; *P* < 0·05). In both groups, erythrocytes count were significantly different from resting values on day 1 of exercise (Table 1; *P* < 0·05). Neutrophils were significantly higher on day 1 for both dietary groups, whereas they remained higher for the control group on day 2 (*P* < 0·05). Monocytes showed a modest, yet significant decrease from resting values on day 1 for both groups (*P* < 0·05). Lymphocytes were significantly lower than resting values in the test diet on day 2 (*P* < 0·05). Eosinophils were significantly decreased from resting on day 2 and 48 h post-exercise in both groups (*P* < 0·05), while test diet dogs had lower eosinophil counts than control diet dogs at 48 h post-exercise (*P* < 0·05). No other significant findings were observed in the CBC due to exercise.

### Serum chemistry and cortisol

Serum chemistry changes were significantly higher from resting values on days 1 and 2 for AST, CK, alkaline phosphatase and phosphorus in both groups, with all but alkaline phosphatase recovering by 48 h post-exercise (*P* < 0·05). Serum potassium was decreased significantly and the serum urea and alanine aminotransferase were significantly decreased in the control group from resting to days 1 and 2 (Table 2; *P* < 0·05). Resting serum albumin was significantly lower than day 1 and 48 h post-exercise (*P* < 0·05). Total protein was significantly lower from resting on day 2 and 48 h post-exercise, but not on day 1 (*P* < 0·05). Globulin was significantly lower than resting on day 2 in the control group only (*P* < 0·05). Serum cortisol was significantly elevated on day 1 only in both groups (*P* < 0·05). AST and CK were elevated in the control group compared with the test diet on day 2 (*P* < 0·01), with AST also being elevated in the control group compared with the test diet group on day 1 (*P* < 0·05). No other significant findings were observed due to exercise or diet.

**Table 2.** Median and ranges of serum chemistry results expressed at rest, after days 1 and 2 of competition, and 48 h after competition in the South Carolina Field Championship in control and supplemented dogs (*n* 5 in each group) Reference ranges are displayed in the left-hand column in parentheses

Serum chemistry (RR)	Group	Resting	Day 1	Day 2	48 h post race
Total protein (5·0–7·4 g/dl)	Control	7·1 (7·0–7·8)	6·6 (6·6–7·2)	6·5 (6·3–6·8)^a^	6·0 (5·6–6·7)^a^
	Test	6·7 (6·1–7·1)	6·6 (5·9–6·6)	6·2 (6·0–6·6)^a^	6·1 (5·9–7·0)^a^
Albumin (2·7–4·4 g/dl)	Control	2·9 (2·7–3·6)	2·7 (2·4–3·1)^a^	2·8 (2·5–3·4)	2·6 (1·9–3·0)^a^
	Test	3·0 (2·4–3·2)	2·8 (2·0–2·9)^a^	2·9 (2·3–3·1)	2·7 (2·3–2·9)^a^
Globulin (1·6–3·6 g/dl)	Control	4·1 (3·5–5·1)	4·1 (3·5–4·8)	3·8 (3·0–3·9)^a^	3·8 (2·8–4·2)
	Test	3·9 (3·1–4·3)	3·8 (3·2–4·4)	3·4 (2·9–4·1)	3·3 (3·2–4·7)
AST (SGOT) (15–66 U/l)	Control	28 (18–37)	112 (102–328)^a,b^	153 (111–313)^a,b^	24 (22–29)
	Test	26 (18–32)	94 (62–110)^a,b^	85 (51–95)^a,b^	22 (18–32)
ALT (SGPT) (12–118 U/l)	Control	22 (19–34)	40 (33–393)^a^	60 (42–324)^a^	56 (40–133)^b^
	Test	31 (25–74)	49 (36–74)	53 (46–69)	61 (38–77)^b^
Alkaline phosphatase (5–131 U/l)	Control	43 (20–55)	79 (48–105)^a,b^	119 (69–171)^a^	78 (69–150)^a,b^
	Test	65 (46–151)	100 (91–241)^a,b^	132 (104–306)^a^	182 (96–216)^a,b^
Urea nitrogen (6–31 mg/dl)	Control	16 (13–20)	28 (17–43)^a^	30 (20–45)^a^	21 (11–34)
	Test	18 (18–33)	25 (23–58)	19 (19–97)	25 (22–72)
Creatinine (0·5–1·6 mg/dl)	Control	0·6 (0·5–0·7)	0·6 (0·6–1·3)	0·6 (0·5–0·8)	0·7 (0·4–0·9)
	Test	0·6 (0·6–1·0)	0·6 (0·5–1·5)	0·6 (0·4–2·1)	0·6 (0·4–1·3)
Phosphorus (2·5–6·0 mg/dl)	Control	5·0 (4·7–6·3)	6·8 (6·5–7·4)^a^	6·7 (6·0–7·2)^a^	5·4 (4·8–5·6)
	Test	5·4 (4·7–5·5)	6·6 (6·2–7·0)^a^	6·2 (5·7–9·0)^a^	4·8 (3·7–7·7)
Glucose (70–138 mg/dl)	Control	81 (72–81)	59 (47–87)	81 (50–86)	81 (64–89)
	Test	87 (72–96)	75 (60–88)	84 (44–97)	79 (56–114)
Calcium (8·9–11·4 mg/dl)	Control	9·7 (7·6–10·5)	9·9 (9·4–10·1)	9·3 (8·8–10·0)	9·1 (8·5–9·8)
	Test	9·8 (9·5–10·3)	9·8 (9·0–10·5)	8·9 (8·5–10·2)	9·9 (8·4–10·0)
Magnesium (1·5–2·5 mEq/l)	Control	1·5 (1·3–2·3)	1·6 (1·5–1·7)	1·6 (1·5–1·7)	1·5 (1·5–1·6)
	Test	1·6 (1·5–1·8)	1·6 (1·5–1·7)	1·6 (1·4–1·9)	1·6 (1·5–2·1)
Sodium (139–154 mEq/l)	Control	144 (143–144)	143 (142–145)	143 (142–151)	146 (145–152)
	Test	144 (144–145)	143 (142–145)	144 (141–147)	145 (141–147)
Potassium (3·6–5·5 mEq/l)	Control	4·8 (4·3–6·9)	4·4 (4·0–4·7)^a^	4·3 (3·9–4·9)^a^	4·5 (4·0–4·7)
	Test	4·8 (4·5–5·4)	4·4 (3·1–5·1)	4·5 (4·2–6·0)	4·6 (4·2–4·8)
Chloride (102–120 mEq/l)	Control	108 (105–110)	110 (106–112)	113 (109–113)	112 (111–119)
	Test	111 (108–115)	112 (106–113)	113 (108–115)	113 (107–114)
Cholesterol (92–324 mg/dl)	Control	263 (222–314)	264 (193–284)	262 (188–296)	271 (147–305)
	Test	304 (263–372)	314 (294–381)	314 (256–340)	300 (259–397)
TAG (29–291 mg/dl)	Control	59 (42–82)	46 (40–52)	59 (42–62)	59 (38–89)
	Test	62 (43–69)	34 (31–50)	98 (34–71)	111 (35–168)
CK (59–895 U/l)	Control	127 (94–215)	1930 (1496–4454)^a^	4020 (2104–12 774)^a,b^	129 (76–182)
	Test	173 (70–215)	1625 (668–1638)^a^	1382 (633–1972)^a,b^	146 (92–187)
Cortisol (1·0–5·0 mg/dl)	Control	2·1 (0·4–3·5)	4·4 (0·5–7·6)^a^	3·5 (1·4–4·3)	2·6 (0·4–3·6)
	Test	1·0 (0·8–3·1)	2·6 (0·5–7·1)^a^	1·8 (0·9–6·0)	1·3 (0·4–2·1)

^a^Indicates a significant difference between resting within group (*P* < 0·05).

^b^Indicates a significant difference between the control and test diet (*P* < 0·05).

### Hunt performance

Of the dogs in the control dietary group, only one dog placed in the category of speed resulting in 1 of 20 possible points. In the test diet group, there were four dogs that placed in three of the four categories with a total of seven placement points out of a possible twenty points. Fisher's exact test revealed a significance in improved performance in the test group (*P* < 0·05).

## Discussion

The results of the present study indicate that hunting dogs behave physiologically more like endurance dogs rather than like sprinting Greyhounds or agility dogs^(^^17^^–^^19^^)^. Biochemical analysis of Greyhounds after racing show increases in packed cell volume, total plasma protein, sodium, potassium, albumin, AST, alanine aminotransferase, CK, alkaline phosphatase and glucose^(^^18^^)^. Conversely, endurance sled dogs show decreases in serum albumin, total protein, sodium, potassium and modest increases in serum phosphorus and chloride^(^^17^^)^. In previous studies, hunting dogs showed no change or increases in serum sodium, potassium, total protein or albumin concentrations; however, these were far shorter duration activities^(^^1^^,^^3^^)^. Our findings show no real sustained changes in serum electrolytes other than a phosphorus increase and potassium decrease, as well as modest decreases in albumin and total protein, similar to changes observed in endurance sled dogs^(^^17^^,^^19^^)^.

Similar to endurance and moderate duration exercise in sled dogs, the blood urea nitrogen, AST, cortisol and CK values were markedly elevated, more than what has been observed in other field hunting or retrieving exercises, which may be a reflection of muscle permeability changes with the duration (5 h a day) or intensity of work^(^^3^^,^^17–^^19^^)^. The serum urea nitrogen alterations were small and likely due to increased protein catabolism, since there were modest decreases in albumin and total protein and the feeding patterns did not change. These data suggest that there was increased energy expenditure and protein catabolism including breakdown of muscle protein for energy. Interestingly, sodium, haematocrit and erythrocyte concentrations did not change, suggesting that haemodilution or haemoconcentration were not factors involved in the modest urea changes. Cortisol elevations were not as robust as expected for the strenuous nature of this exercise bout when compared with sprinting sled dogs^(^^20^^)^; however, the rises in cortisol were higher than observed in endurance sled dogs^(^^19^^)^. The results must be interpreted cautiously due to the modest delay (up to half an hour) in blood collection after days 1 and 2 of the event which can affect overall results in cortisol analysis^(^^20^^)^.

Nutritionally, there have been no studies confirming that supplementation with antioxidants or other performance enhancing agents in dogs can lead to decreased CK and AST parameters post-performance^(^^8^^–^^10^^)^. Although both groups had elevated AST and CK values, the treated dogs' serum concentrations were significantly lower than the control dogs on day 2 for CK and on both days 1 and 2 for AST. This may reflect decreased muscle cell permeability and damage over the duration of the event. However, as this was not a blinded study, selection of the treatment group may have been biased towards treating more successful dogs. Harder working dogs should have had greater elevations in CK and AST, yet the treated dogs displayed lower CK and AST concentration on day 2 of the trial. It is possible that the treated dogs experienced less muscle stress in the hot environment due to the supplement.

The performance of the test group *v.* the control group in the hunt is provocative and is contradictory to the CK values. During the hunt test, dogs are evaluated and scored based on four categories, including endurance, speed, tracking and overall placement within the hunt. In the treatment group, four of five dogs received points while in the control group, only one placed in any category which was significant. Interpretation of this finding may be a reflection of handler bias in dog selection for supplementation. Based on discussions with the handlers, dogs were treated in groups within pens based on dog compatibility, rather than being fed individually. Since dogs were not housed based on performance, this bias is less likely.

The action of the supplement to mitigate muscle permeability changes appears significant and may be due to the additional l-carnitine and betaine (see Supplementary Table 1), as well as other vitamin and mineral additions. When examining the constituents in the premix, studies have shown increased antioxidant capacity in serum or tissue; however, they have never been shown to alter muscle metabolism, energetics or permeability as measured by alterations in CK. Betaine has been shown to improve intracellular pH and osmolality and has been correlated to improved heat tolerance in agricultural animals and exercise performance in human athletes^(^^5^^,^^6^^)^. Betaine is traditionally used as a choline substitute in animal feeds, but has not been evaluated as a performance enhancing supplement for dogs.

Overall, this study reflects new information regarding the physiological changes associated with endurance activities in hunting dogs during field events. The changes observed are similar to endurance sled dogs rather than sprinting dogs (Greyhounds and agility dogs). Surprisingly, the use of a proprietary performance enhancing supplement showed improved CK and AST values and hunt test placement in the treated dogs. The results of the present study are interesting; however, due to the study design they are not conclusive. Further work with this supplement in a larger group of dogs using a blinded crossover study design would be ideal to prove that the proprietary blend of nutrients improved performance and improved muscle cell permeability.

## Supplementary material

The supplementary material for this article can be found at http://www.journals.cambridge.org/jns
